# Clinical Findings of Pediatric HIV Infection in a Tertiary Center in Turkey

**DOI:** 10.4274/balkanmedj.2015.1571

**Published:** 2017-05-15

**Authors:** Murat Sütçü, Manolya Acar, Hacer Aktürk, Selda Hançerli Torun, Hayati Beka, Ali Ağaçfidan, Nuran Salman, Ayper Somer

**Affiliations:** 1 Department of Pediatric Infectious Diseases, İstanbul University İstanbul School of Medicine, İstanbul, Turkey; 2 Department of Clinical Microbiology, İstanbul University İstanbul School of Medicine, İstanbul, Turkey

**Keywords:** child, HIV infection, Turkey

## Abstract

**Background:**

Paediatric HIV infection is different from the adult type of disease in many ways, including transmission routes, clinical findings and treatment strategies.

**Aims:**

To evaluate clinical data of paediatric patients with HIV disease.

**Study Design:**

Retrospective cross-sectional study.

**Methods:**

The charts of 22 paediatric patients diagnosed with HIV infection in our clinic during a 14 year period through 2001-2015 were retrospectively analysed. Clinical data, laboratory findings, treatment modalities and outcomes were recorded.

**Results:**

The mean age of diagnosis 61.9±49.2 months and the mean follow-up period was 60.3±37.5 months. Seven patients (31.8%) were foreigners and the most common transmission route was vertical transmission (n=16, 72.7%). The most common presenting symptom and the sign were history of recurrent upper respiratory tract infections (n=8, 36.4%) and lymphadenopathy (n=12, 54.5%), respectively. Recurrent pneumonia (n=6, 27.3%), prolonged fever (n=5, 22.7%), recurrent otitis media (n=4, 18.2%), and gastroenteritis (n=4, 18.2%) were other clinical symptoms. Other than bacterial sinopulmonary infections, tuberculosis was the most frequent opportunistic infection (n=3, 13.6%). Mortality occurred in two patients (9.1%).

**Conclusion:**

Although mostly vertically transmitted, HIV infection may be diagnosed throughout the childhood. Frequently encountered signs and symptoms may be the reason for doctor admission. High clinical suspicion together with detailed anamnestic data and physical findings constitute the basis for pediatric HIV diagnosis.

Worldwide, thirty five million people are infected with HIV, with approximately 3.2 million (9%) of them being under the age of 15 (1). According to the Turkish Ministry of Health report in June 2015, 10.475 patients with the diagnosis of HIV/AIDS are living in our country (2). Although the incidence of newly diagnosed HIV/AIDS patients has dropped by 58% in children and 38% in total, the number of recent cases has been rising (3). In the first half of 2015, 893 new cases were diagnosed, with 22 of them in patients below 19 years of age (2).

Because mother to child transmission is the main mechanism of paediatric HIV acquisition, most children present in infantile period. Nevertheless, the age of presentation may be variable. Several signs and symptoms including recurrent bacterial infections, prolonged fever, persistent diarrhoea, frequent thrush, generalised lymphadenopathy, chronic parotitis and delay in development should warn the clinicians of possible HIV infection.

With the introduction of highly active antiretroviral treatment (HAART), HIV/AIDS infection which was once believed to be a rapidly lethal disease has become a chronic, progressive disorder with increased life expectancy (4). Due to its rarity, data in the literature regarding paediatric HIV infection do not go beyond case reports in our country. To be able to create our national database, it is very important to evaluate the epidemiological and clinical characteristics of HIV-infected children in our country. In order to contribute our experience, we hereby document the clinical findings of our paediatric HIV/AIDS patients.

## MATERIALS AND METHODS

This study is a clinical analysis of 22 paediatric patients diagnosed with HIV infection in our paediatric infectious disease clinic during a 14 year period from 2001-2015. For children ≥18 months, HIV infection diagnosis is based on positive HIV antibody testing confirmed by a second HIV antibody test and a positive virological test. HIV diagnosis is established by positive virological test for HIV or its components confirmed by a second virological test obtained from a separate determination taken more than four weeks after birth for smaller children (<18 months) (5). Classification of the patients was performed according to the clinical staging and case definition of HIV developed by the World Health Organisation (WHO) in 1990 and revised in 2007. Opportunistic infections (OIs) were those used to define WHO clinical stages 2, 3 and 4 (6).

The charts of the patients were retrospectively scanned in order to gather epidemiologic, clinical and laboratory data, treatment modalities, treatment response, side effects, complications and outcomes. Laboratory investigations consisted of complete blood count, serum immunoglobulin levels, CD4 and CD8 counts, and polymerase chain reaction for HIV RNA from all participants. Cranial magnetic resonance investigation (MRI) reports of patients with neurological symptoms were evaluated.

Serum immunoglobulin measurements have been analysed nephelometrically (Date Behring Marburg GMBH, Germany). CD4PE, and CD8APC monoclonal antibodies have been measured by four-color flow cytometry method (BD Facs Calibur, BD Calibur, BD Biosciences, San Jose, California, USA). Patient data were compared with reference values adjusted for age (7).

In our Microbiology Department, HIV RNA analysis was performed by the LCx HIV RNA Quantitative assay (Abbott Laboratories, North Chicago, III, US) with a dynamic measurement range <178-5.011.872 copies/mL between 2001 and 2007, by an Artus HIV-1 QS-RGQ test (QIAGEN Ltd, Crawley, UK) with a dynamic measurement range <72-25.000.000 copies/mL from 2007 to 2010, and by COBAS^®^ AmpliPrep/COBAS^®^ TaqMan^®^ HIV-1 test 96 system (Roche Molecular Diagnostics, Basel, Switzerland) with a dynamic measurement range of 20-10.000.000 copies/mL since 2010.

Our study was approved by the Clinical Trials Ethical Committee with the number 2015/2065. Parents of all study participants were informed about the study and their informed consent was taken.

### Statistical analysis

Statistical analysis of data was performed with statistical package for social science (SPSS) for Windows version 21.0 (SPSS Inc.; Chicago, IL, USA). Normality was assessed by Shapiro-Wilk tests. Data are presented as median, minimum, maximum, frequency and percentage.

## RESULTS

### Clinical information

Twenty two paediatric patients, 9 male (40.9%), with the mean age of diagnosis 61.9±49.2 months and mean follow-up period of 60.3±37.5 months were enrolled in our study (Table 1). When patients are separated according to age of presentation, 5 patients (22.7%) were below 1 year of age, whereas 7 patients (31.8%) aged between 1-5 years and 10 patients (45.4%) were ≥6 years.

Three patients (13.6%) were asymptomatic initially and diagnosed as stage 1 HIV infection according to the WHO clinical classification system. One patient (4.5%) was diagnosed as stage 2, while 5 (22.7%) and 13 (59.1%) were diagnosed as stage 3 and 4 HIV infection, respectively.

Fifteen (68.1%) patients were Turkish citizens. Five patients (22.7%) were from Uzbekistan, 1 patient (4.5%) was from Romania and the other was from Russia. Both parents of 11 patients (50%) were infected with HIV. Five patients (22.7%) had only a HIV positive mother, and 3 patients (13.6%) had siblings with HIV infection.

Vertical transmission (VT) was the most common route, being encountered in 16 patients (72.7%). Among those, 9 patients (40.9%) were born via vaginal delivery. Three children (13.6%) had history of blood transfusion during an operation, 2 patients (9.1%) had dentistry applications and no possible route could be obtained for the other patient.

### Presenting signs and symptoms

The most common presenting symptom was history of recurrent upper respiratory tract infections (URTI), which was encountered in 8 patients (36.4%) (Table 2). The most common clinical findings were lymphadenopathy and various kinds of dermatological manifestations (diaper dermatitis n=2, eczematous skin lesions n=2, non-specific maculopapular rash n=2, vesicular eruption n=1, widespread molluscum contagiosum n=1, persistent oral monoliasis n=1, scabies n=1) were observed in 12 patients (54.5%).

Recurrent pneumonia (n=6, 27.3%), prolonged fever (n=5, 22.7%), recurrent otitis media (n=4, 18.2%), recurrent gastroenteritis (n=4, 18.2%), weight loss (n=1, 4.5%), recurrent parotitis (n=1, 4.5%), pancytopenia (n=1, 4.5%) and isolated thrombocytopenia (n=1, 4.5%) were the other presenting symptoms. One patient (4.5%) was admitted with the features of wasting syndrome. Growth retardation was observed in 3 (13.6%) patients. Eleven patients (50%) had splenomegaly, 10 patients (45.5%) had hepatomegaly and 4 patients (18.2%) had pathological breath sounds.

### Opportunistic infections

Seventeen patients (77.2%) with a median age of 38 months (range, 1-165 months) presented with OIs. Median CD4% among patients with OIs was 10% (range, 1-38%) with average viral load (VL) of 366.000 copies/mL (range, 200-4.378.407 copies/mL). The most common OIs were recurrent bacterial sinopulmonary infections (SPI) such as URTI (n=8, 36.4%), pneumonia (n=6, 27.3%) and otitis media (n=4, 18.2%). Tuberculosis (TB) was the most frequent OI (n=3, 13.6%) other than recurrent SPI. Chronic herpes infections (n=2, 9.1%), cryptococcus septicaemia (n=1, 4.5%), disseminated molluscum contagiosum (n=1, 4.5%) and disseminated cytomegalovirus infection (n=1, 4.5%) were also observed. The incidence of OI decreased to 9.1% (n=2) at the evaluation of last patient visit. Both patients showed an incompatibility with treatment and were lost to follow-up for a reasonable time before being readmitted with high VL.

### Laboratory data

Eight patients (36.3%) presented with anaemia and lymphopenia, 7 patients (31.8%) had neutropenia and 3 patients (13.6%) had thrombocytopenia initially. Eosinophilia was encountered in 3 patients (13.6%). Mean IgG level was 1746.7±1087.1 gr/dL and 11 patients (50%) had increased serum IgG levels. Median IgA and IgE levels were 128.5 (2-810) gr/dL and 45 (5-59.300) kU/L, respectively and 8 patients (36.3%) had increased serum IgE levels.

Median CD4% at the start of antiretroviral (ART) was 13% (range, 1-68%). Eight patients (36.4%) had CD4% below <10%. Median VL was 297.047 (range, 200-4.950.000 copies/mL). HIV RNA level was >10.000 copies/mL in 81.8% of the cases. At last patient visit, median CD4% and VL were 31% (range, 12-41%) and <20 copies/mL (range, <20-136.000) respectively. Sixteen patients (72.7%) had undetectable (<20 copies/mL) VL.

### Treatment and outcomes

All of our patients were started on HAART according to the Turkish Ministry of Health HIV/AIDS guideline 2013 recommendations (8). The initial ART choice was protease inhibitor-based regimen (lamivudine + zidovudine + lopinavir/ritonavir) for all patients. During the follow-up period, treatment modification was applied to 6 patients (27.3%) due to drug side effects (n=3, 13.6%), drug resistance (n=2, 9.1%) and co-infection of HBV and HCV in one patient (Table 3). The mean duration for ART to achieve undetectable VL was 7.0±1.5 months.

All patients were given prophylactic trimethoprim sulphamethexasole; in addition, 15 patients (68.1%) had isoniazid, 7 patients (31.8%) had fluconazole and 2 patients (9.1%) had acyclovir prophylaxis. Fifteen patients (68.1%) showed full compliance to treatment while 5 patients (22.7%) had problems during treatment and two patients (9.1%) were totally incompatible. Adverse drug reactions were observed, such as thrombocytopenia (n=2, 9.1%), megaloblastic anaemia (n=1, 4.5%) and neutropenia (n=1, 4.5%).

Although none of the patients presented with a neurological system abnormality at the beginning, HIV related encephalopathy was encountered in 2 patients (9.1%) during the follow-up period. Cranial MRI revealed periventricular leukomalacia in 1 patient (4.5%), and the other was compatible with ischaemic findings. Splenectomy was performed in one patient for persistent thrombocytopenia.

The incidence of malignancy was 4.5%. The patient was a 16 year-old girl diagnosed with HIV infection and Non-Hodgkin lymphoma, concomitantly. She passed away because of relapse after initial successful regression. A four month old girl had died secondary to progressive lower respiratory tract infections. The total mortality rate was 9.1% (n=2), for the above-mentioned reasons.

## DISCUSSION

By 2013, there have been 240.000 recent paediatric HIV-infected events under age of 15 (1). Since more than 90% of children acquire HIV infection by way of VT; therefore, the eradication of paediatric cases can only be achieved by precaution methods generally known as prevention of mother to child transmission (PMCT). As a result of PMCT efforts, the number of new congenital HIV infection cases dropped from 1650 to 107 (from 1991-2013) in the USA (9). The Treat Asia Paediatric HIV observational database reported perinatal exposure incidence to be 94.1% (10). Children acquiring HIV infection through blood products (1.1%), sexual intercourse/abuse (0.5%) and other causes (4.3%) was extremely rare. Although the ratio was smaller, VT was also the most common route for transmission in our study (72.7%). HIV exposure by blood transfusion was responsible for 3 patients (13.6%) while 2 patients (9.1%) had a history of dentistry application. None of those were Turkish citizens. A possible route of transmission could not be determined in one patient (4.5%).

In the scope of PMCT interventions in our country, combined ART for all HIV-infected pregnant women independently from virological, immunological or clinical status, elective Caesarean section at the 38^th^ gestational week for patients with VL of >1000 copies/mL, zidovudine therapy to both pregnant women perinatally and to the newborn immediately after birth and cessation of breastfeeding are strongly recommended (9). According to the Turkish Ministry of Health report in 2014, the rate of mother to child transmission (MCT) is reported to be 0.6%. That is far below the data obtained from European countries, with an incidence of 1.2-1.4% reported (11,12,13). Nevertheless, this ratio seems to be underestimated since the incidence of unknown exposure is 56.7% (2). In a previous study, the rate of MCT was found to be 6.2% (14). However, the number of patients in that study was not enough for convenient interpretation, and thus we believe that the real ratio is somewhere between the two (0.6-6.2%).

Recurrent SPIs, chronic diarrhoea, and persistent oral monoliasis are frequently encountered in HIV-infected children. Merchant et al. (15), in their study including 285 paediatric HIV/AIDS patients, reported the incidence of recurrent/chronic diarrhoea, monoliasis, prolonged fever and lower respiratory tract infection to be 15%, 14.7%, 12.6% and 8.4%, respectively, supporting our findings that the most common symptom is the presence of recurrent SPI.

HIV/AIDS patients can present with variable skin and mucosal lesions which may or may not be relevant to infectious disorders; the incidence may be as high as 79% (16). Consistent with previous reports, various dermatological findings including non-specific maculopapular rash, diaper dermatitis, eczematous skin lesions, molluscum contagiosum lesions, vesicular eruption, persistent monoliasis and scrabies were encountered in 54.5% of our study patients (Table 2). While, Kaposi sarcoma, cryptococcal and human papilloma virus related lesions are encountered in patients with low CD4 levels, the ones with good immunological status may have seborrheic dermatitis or kseroderma (17). Molluscum contagiosum, one of the frequent viral eruptive diseases of children, when it is chronic and generalised, may be a sign of underlying immune deficiency disorders, like HIV infection (18). One of our patients presented with generalised molluscum infection with the absolute CD4 value being 42 cells/mm^3^ (2%). The other patient, an 8 year old boy with an absolute CD4 count of 6 cells/mm^3^ (1%) and serum IgE level of 59.300 kU/L, had generalised pruritic skin lesions and was diagnosed with scabies.

Somatic growth retardation and malnutrition prevalence among HIV infected children can be as high as 83.3%, especially in children with HIV endemic areas (15). Similarly, a 2003 study from South Africa (19) and a 2001 study from Ethiopia (20) reported the prevalence of severe protein energy malnutrition to be 34.3% and 22%, respectively. These data could be explained by the high incidence of malnutrition among the HIV non-infected population at the same time. Our study revealed growth retardation in 3 children (13.6%), far below the reported studies. The prevalence of other physical findings such as lymphadenopathy and hepatosplenomegaly were similar to previous data (19,20,21).

Although dramatic declines have been observed in the incidence of OIs both in children and adults with the ascending use of HAART, the spectrum of OIs and their frequencies have not changed in recent years (5). Recurrent URTI, persistent monoliasis and TB are still the most frequent. Recurrent SPI (n=18, 81.8%) and TB (n=3, 13.6%) were also the most common in our study. The average CD4% among our patients with OIs were 10% (range, 1-38%), initially. TB can disseminate, may reactivate or progress rapidly secondary to immunosuppressive nationality of HIV (22,23,24). WHO estimates the HIV prevalence to be 10-60% among children with TB and mortality in the case of co-infection is reported to be 20-35% (25). That is 6 times greater than that in patients with HIV infection alone (26,27). Although HAART, the degree of immune suppression and HIV replication status may alter the incidence of TB co-infection, the occurrence of TB in children with high CD4 values under HAART suggests a multifactorial process (28). In our cohort, 3 patients (13.6%) were diagnosed with the co-infection of TB (two patients with miliary, and one patient with pulmonary TB). Miliary TB were encountered in patients with high replication load and low CD4% (HIV RNA, 443.000 and 1.160.000 copies/mL and CD4% as 10% and 6.6%, respectively). Pulmonary TB was observed in a 5 year old congenital HIV infected patient during ART with CD4% above 25%.

During the course of HIV infection, neurological system involvement can be seen secondary to OIs, malignancies, inflammatory processes or the disease itself. The incidence is reported to be 11-57.5% (29). HIV related cognitive and motor functional abnormalities are mainly encountered in patients with CD4 levels >200 cells/mm^3^. In the case when immune suppression is apparent (CD4 <200 cells/mm^3^) AIDS-related tumours, central nervous system lymphoma and OIs are the most important aetiological factors (30). Two patients (9.1%) demonstrated neurological system abnormalities during the follow-up period in our cohort.

Haematological system abnormalities are multifactorial. Each of the major cell lineages may be affected during the course of disease. These cytopenias may be associated with effects of infection, inflammation, HIV-related malnutrition, malignancy or side effects of ART (31). The most common haematological abnormality, anaemia, neutropenia and thrombocytopenia incidence are reported to be 20%, 10% and 10%, respectively (32). Particularly, CD4 lymphopenia with reversed CD4/CD8 ratio should raise the suspicion of HIV infection. In our study, 8 patients (36.3%) had lymphopenia at the time of diagnosis. Neutropenia (n=7, 31.8%), anaemia (n=8, 36.3%), and thrombocytopenia (n=3, 13.6%) incidence were similar to the data in the literature.

With the introduction of HAART, paediatric HIV infection has switched from a highly lethal disease to a chronic disorder with prolonged life expectancy. Unlike adult HIV/AIDS, indications for paediatric HAART have a broader spectrum. The Turkish Ministry of Health 2013 HIV/AIDS guideline absolutely recommends the treatment of all HIV-infected children <1 year of age regardless of immunological, virological or clinical status. For children beyond infancy, in the presence of AIDS-defining symptoms, high VL (>100.000 copies/mL) or low CD4 levels (<25%), treatment is strongly recommended. For children older than one year of age with minimal or no symptoms, treatment should be preferred (8). Based on our national guidelines, in our clinic, all children with definite HIV diagnoses have been started on ART. Our initial cART choice was 2 NRTI +1 PI (lamivudine + zidovudine + lopinavir/ritonavir). In our study group, the average time to achieve undetectable VL was 7.0±1.5 months. This was an acceptable time period. Hence, we can strongly recommend this regimen at the start of ART for our country. ART-related cytopenia, dyslipidaemia, hepatotoxicity, and pancreatitis are the common side effects which may require alterations in therapy. Thrombocytopenia (n=2, 9.1%), megaloblastic anaemia (n=1, 4.5%) and neutropenia (n=1, 4.5%) were obtained as drug side effects in our study. One patient with resistant thrombocytopenia, after undergoing corticosteroid and IVIG trial several times, underwent splenectomy.

The incidence of malignancy, mainly non-Hodgkin lymphoma, Kaposi sarcoma and leiomyosarcoma, among children with HIV/AIDS is increased, as reported in adults (33). There are limited data regarding malignancy rates of HIV-infected children after HAART. Kest et al. (34), in their study including 2969 paediatric HIV/AIDS patients, reported the significant decrease of malignant tumours after two years of ART. In our study population, a sixteen year-old girl presented with generalised lymphadenopathy. She was diagnosed concomitantly with HIV infection and Burkitt lymphoma. After successful treatment, she passed away due to the relapse of disease. The other mortality case in the course of our follow-up was a 4 month-old patient with congenital HIV infection who died secondary to lower respiratory tract infection.

This study has several limitations. It is a retrospectively designed study based on a small number of cases due to rarity of pediatric HIV infection in our country. It reports evolution of clinico-laboratory findings of these cases followed by a peadiatric HIV center in Istanbul, therefore it lacks statistical studies apart from those describing demograpics, clinical and laboratory data.

In conclusion, pediatric HIV infection is a generally feared and missed clinical condition due mainly to unfamiliarity. Literature search reveals pediatric data mostly from HIV endemic, resource limited countries. There are several studies from our country involving adult population, which differ from pediatric HIV in some aspects. This study is the first report in our country that describes the clinical experience of a tertiary pediatric HIV center with the aim of to raise awareness about pediatric HIV.

## Figures and Tables

**Table 1:**
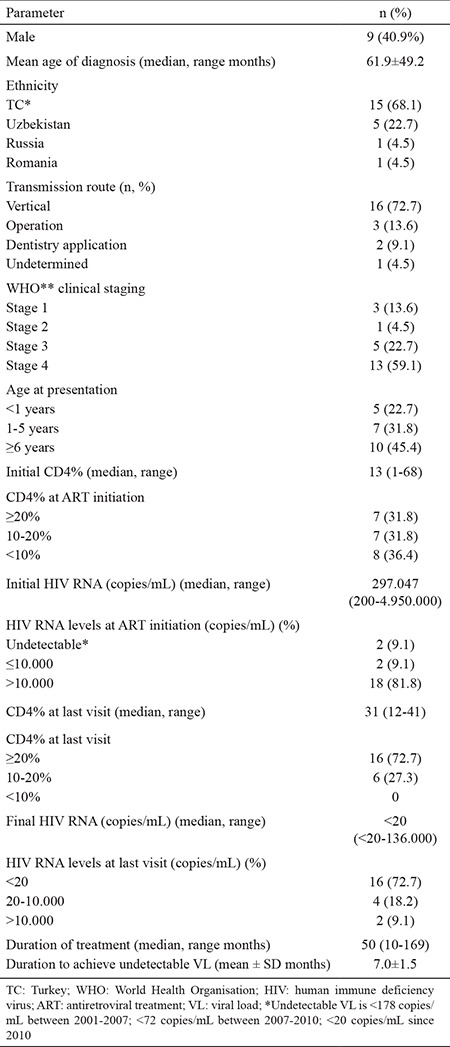
Characteristics of HIV infected children

**Table 2:**
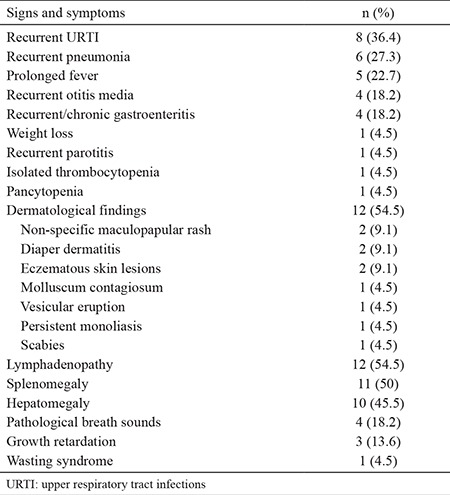
Clinical presentation of patients

**Table 3:**
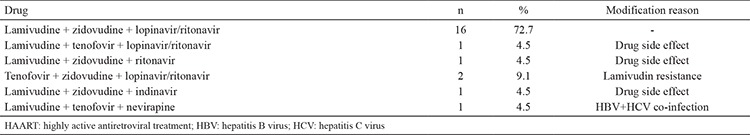
HAART regimen at last patient visit
